# Examining a model of anxiety in autistic adults

**DOI:** 10.1177/13623613231177777

**Published:** 2023-06-16

**Authors:** Saskia Riedelbauch, Sebastian B Gaigg, Tobias Thiel, Veit Roessner, Melanie Ring

**Affiliations:** 1 Klinik und Poliklinik für Kinder- und Jugendpsychiatrie und –psychotherapie, Medizinische Fakultät, Technische Universität Dresden, Dresden, Germany; 2 City, University of London, UK

**Keywords:** alexithymia, anxiety, autism, emotion regulation, intolerance of uncertainty, sensory processing

## Abstract

Anxiety disorders commonly occur in autism. Existing studies implicate intolerance of uncertainty, alexithymia, sensory processing differences and emotion regulation difficulties as influencing factors of anxiety in autism. To date, a few studies have considered the combination of these factors within the same sample. This study used structural equation modelling to test the prediction that intolerance of uncertainty and emotion regulation constitute more direct causes of anxiety in autism that mediate the influences of sensory processing difference and alexithymia as more sequential contributing factors. Autistic (*n* = 86) and non-autistic adults (*n* = 100) completed a battery of self-report questionnaires. Only when applied to each group separately, the broad predictions of the model were confirmed for the autistic group following data-driven additions of paths between sensory processing difference and anxiety and alexithymia implying that sensory processing difference contribute indirectly as well as directly to individual differences in anxiety. For the non-autistic group, model fit could only be achieved after removing autism-related traits and sensory processing differences as predictors of anxiety. These results suggest that aetiology and expression of anxiety in autism partially overlap with what is observed in the general population except that sensory processing differences appear to play a relatively unique role in the context of autism.

**Lay abstract:**

Anxiety disorders are common in autism. Research studies have identified factors that influence anxiety in autism, such as difficulties with uncertain situations, difficulties understanding own emotions, differences in processing sensory input (related to our senses) and difficulties regulating emotions. To date, a few studies have considered the combination of these factors within the same sample. This study used structural equation modelling to test the contribution of these factors in autism. Autistic (*n* = 86) and non-autistic adults (*n* = 100) completed a battery of self-report questionnaires. Only when applied to each group separately, the broad predictions of the model were confirmed for the autistic group. The model confirmed that difficulties with uncertain situations and in regulating emotions play a central role in anxiety in autism. Difficulties understanding own emotions and differences in processing sensory input both contribute to anxiety indirectly through their respective interrelation with the other two factors (difficulties with uncertain situations and in regulating emotions). Importantly, the results imply that sensory processing differences contribute not only indirectly but also directly to individual differences in anxiety. For the non-autistic group, model fit could only be achieved after removing autism-related traits and sensory processing differences as predictors of anxiety. These results suggest that cause/development and expression of anxiety in autism partially overlap with what is observed in the general population except that sensory processing differences appear to play a relatively unique role in the context of autism.

Autistic individuals commonly experience co-occurring anxiety disorders, with a life time prevalence of up to 42% (Hollocks et al., 2019). A recent study suggests that young autistic adults have a risk of an anxiety diagnosis two and a half times higher compared with non-autistic persons from the general population, with autistic individuals without intellectual disability (ID) appearing to have the highest risk (Nimmo-Smith et al., 2020). In combination with aspects of the defining clinical characteristics of autism (difficulties in social interaction, communication and repetitive interests and behaviours; American Psychiatric Association, 2013), anxiety disorders negatively impact upon quality of life and are, therefore, an important target for interventions (van Steensel et al., 2012). Despite an emerging consensus about the factors that contribute to high levels of anxiety in autism, questions remain about how multiple factors interact in the development and maintenance of anxiety in autism.

Existing research has identified alexithymia (ALX), emotion regulation difficulties, intolerance of uncertainty (IU) and sensory processing differences as some of the most significant contributors to anxiety in autism (Cai et al., 2018; Glod, 2017; Lidstone et al., 2014; Maisel et al., 2016; Pickard et al., 2020; South & Rodgers, 2017; Wigham et al., 2015). ALX describes a trait characterised by difficulties identifying and describing one’s own emotional states (cf. Wittchen & Hoyer, 2011). Compared to around 10% in the general population, about 50% of autistic individuals report high levels of ALX (Kinnaird et al., 2019), and there is consistent evidence for a link between ALX and anxiety in both autistic as well as non-autistic individuals (Milosavljevic et al., 2016; Oakley et al., 2020). *Emotion regulation* (ER) comprises all the strategies people use to modulate their emotions in order to reach personal goals (Thompson, 1994). The literature on ER distinguishes between maladaptive (e.g. suppression) and adaptive (e.g. reappraisal) strategies on the basis of their longer-term influences on mental health and wellbeing (see, for example, Gross & John, 2003). Autistic individuals tend to use adaptive strategies less often and maladaptive strategies more often (Samson et al., 2012). This pattern presents a well-established risk factor for anxiety in autism (White et al., 2014) and has also been linked to ALX (Samson et al., 2012). The concept of IU is defined as the tendency to experience uncertain situations as aversive and, thus, avoid them. The construct was first identified as an important contributing factor to the development and maintenance of generalised anxiety in the general population (Dugas et al., 1998) and a considerable literature now demonstrates that IU is significantly elevated in autism where it consistently predicts higher levels of anxiety (Boulter et al., 2014; Hwang et al., 2019; Jenkinson et al., 2020; Maisel et al., 2016). Finally, *sensory processing differences* (SPDs) such as hypo- or hyper-sensitivity to sensory stimuli, which are now considered a defining characteristic of autism (American Psychiatric Association, 2013), have also been linked to anxiety in a number of studies (Gillott & Standen, 2007).

Although the links between each of the constructs outlined above (ALX, ER, IU and SPD) and anxiety have been well established in autism, questions remain about their relative and unique contributions as well as their causal relations. For example, South and Rodgers (2017) suggest that SPD and ALX could lead to anxiety relatively indirectly by rendering external and internal sensory experiences less predictable and comprehensible, thereby contributing to greater levels of IU. This suggestion has received support from a number of studies which have demonstrated that IU mediates the relationship between SPD and anxiety (Hwang et al., 2019; Wigham et al., 2015), as well as between ALX and anxiety (Moore et al., 2021; Ozsivadjian et al., 2021) in autism spectrum disorder (ASD). However, Pickard et al. (2020) recently found that SPD and IU could independently predict individual differences in social anxiety in autistic youth, and Neil et al. (2016) have shown that the interrelations among SPD, IU and anxiety can also be understood under alternative causal assumptions. Rather than considering SPD as a contributing cause of anxiety, the authors show that anxiety can also be considered a mediator of the relationship between IU and SPD on the basis that anxiety resulting from IU might lead to hypervigilance and, thus, hypersensitivity to the sensory environment (see Green & Ben-Sasson (2010) and Normansell-Mossa et al. (2021) for further discussion).

In addition to debates concerning the interplay among SPD, IU and anxiety, findings concerning the interactions among IU, anxiety, ALX and ER are also not entirely consistent. For example, Maisel et al. (2016) found that ALX, alongside emotional acceptance (an adaptive ER strategy often associated with mindfulness-based practices), fully accounted for higher levels of anxiety in autistic adults, with IU explaining no further individual differences in anxiety over and above these factors. Morie et al. (2019) also found that ALX and ER fully mediated the relationship between core autism traits and anxiety, although IU was not measured in this study. By contrast, Pickard et al. (2020) found that ALX played no independent role in predicting social anxiety once IU and SPD were controlled for, and Cai et al. (2018) found that IU fully mediated the relationship between ER difficulties and anxiety in autistic adolescents and young adults. Finally, a recent study of autistic adults with ID by Sáez-Suanes et al. (2020) showed that both IU and ER played independent mediating roles between autism-related behavioural traits and anxiety.

As the overview above illustrates, despite a clear consensus concerning some of the key contributing factors to anxiety in autism, the precise nature of their interactions remains unclear, in part because very few studies have considered all of the relevant factors in the same sample. In fact, to the best of our knowledge, only the study by Pickard et al. (2020) has done so to date. This study was specifically concerned with the influences of ALX, SPD, IU and ER on social anxiety in autism, and the analyses were somewhat limited to examining binary associations and simple mediations. Nevertheless, such studies are critical for the development and further refinement of theoretical models such as the one proposed by South and Rodgers (2017), which will ultimately be important for guiding clinical practice as well as much needed longitudinal research in this area. This study, therefore, builds on the work outlined above with the aim of testing some key predictions that emerge from the literature to date. Specifically, we tested the validity of the theoretical model outlined in Figure 1, which represents an extension of the model proposed by South and Rodgers (2017) based on the combination of findings outlined above. Specifically, this model predicts that IU and ER constitute the most proximal causes of anxiety in autism, with SPD and ALX contributing more indirectly as sources of greater IU and ER.

**Figure 1.**
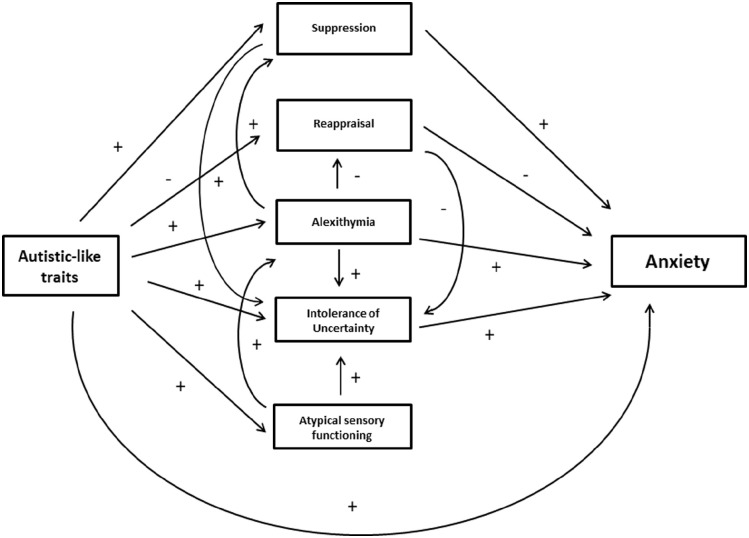
Hypothesised model of the relationships between autism and anxiety and their influencing factors. *Note*. ER in this model is operationalised as a ratio of adaptive (reappraisal) and maladaptive (suppression) strategy use whereby a greater ratio indicates a more maladaptive pattern of emotion regulation. Thus, all predicted associations in this model are positive (+).

## Methods

### Sample

A sample of 186 adults was studied, consisting of 86 autistic adults aged 18–67 years (21 female, 65 male, M_age_ = 33.5 years, SD_age_ = 13.3) and 100 non-autistic adults aged 18 to 70 years (33 female, 67 male, M_age_ = 33.9 years, SD_age_ = 13.9). This group consisted of 87 adults without any psychological disorder (NA) and 13 adults with anxiety disorders (ANX). Distributions of the highest educational degree did not differ between groups (*U* = 3730.00, *p* = .104). Also, there were no significant differences in age (*t*(184.00) = 0.19, *p* = .849, Cohen’s *d* = 0.03) or gender (χ^2^(1) = 1.65, *p* = .20) between groups. Therefore, all following analyses did not further consider these variables. Basic demographic information is presented in Table 1.

**Table 1.** Descriptive statistics of participants.

	Autistic (*n* = 86)		Non-autistic (*n* = 100)	
Characteristic	*n*	%	*n*	%
Gender				
Female	21	24.4	33	33.0
Male	65	75.6	67	67.0
The highest educational degree				
No degree	4	4.7	0	0.0
Degree after 9 years of school	5	5.8	6	6.0
Degree after 10 years of school	37	43.0	31	31.0
Higher education entrance qualification (after 12 years of school)	17	19.8	34	34.0
University or college degree	19	22.1	27	27.0
Postgraduate degree	4	4.7	2	2.0
Additional measures for autistic sample		*N* (autistic)	M (SD)	Range
FIQ^a^		71	103.9 (15.6)	75–145
ADOS COM^b^		73	4.1 (1.9)	0–8
ADOS SA^c^		73	7.8 (3.3)	2–11
ADOS CREA^d^		72	1.2 (0.7)	0–2
ADOS RRB^e^		72	1.1 (1.1)	0–4

*Note.* The highest educational degree according to the German education system. Section two: information was not available for all autistic participants.

a Full-Scale Intelligence Quotient assessed by HAWIK-IV, WIE, WAIS-IV, HAWIE-R, HAWIK-III and CFT1.

b ADOS Communication subscale.

c ADOS Reciprocal Social Interaction subscale.

d ADOS Creativity subscale.

e ADOS Restricted and Repetitive Behaviours subscale. Higher scores on ADOS subscales indicate higher symptom level of the autistic spectrum.

Participants took part from May 2020 to May 2021. Autistic participants were patients of the Autism Clinic of the University Hospital Carl Gustav Carus of Technische Universität Dresden, Germany and were recruited via telephone, email, mail or through their therapists. We included only individuals with an intelligence quotient (IQ) of 75 or above in order to ensure comprehension of all relevant self-report questionnaires. Autistic participants had a clinical diagnosis of childhood autism (F84.0) or Asperger’s syndrome (F84.5) according to the *International Classification of Diseases* 10 (ICD-10, World Health Organization, 2016). The formal diagnosis was obtained prior to participation through clinical assessments with the German versions of the Autism Diagnostic Observation Schedule 1 or 2 (ADOS; Poustka et al., 2015; Rühl et al., 2004) and the Autism Diagnostic Interview–Revised (ADI-R; Bölte et al., 2006). Full-scale IQ was assessed with the German adaptations of the Wechsler Intelligence Scale for adults (Petermann, 2012; Tewes, 1991; Von Aster et al., 2006) and for children (Petermann & Petermann, 2007; Tewes et al., 1999) or the Cultural Fair Intelligence Test Scale 1 (Weiß et al., 1997) shortly after the individuals’ admission to the Autism Clinic (see Table 1 for IQ and ADOS test results). Autistic participants had a mean age of diagnosis of 23.9 years (SD = 15.5). Following comprehensive clinical evaluations by a multidisciplinary team at the University Hospital, 41.9% of autistic participants presented one and 31.4% had two or more co-occurring psychological disorders according to ICD-10 (lifetime F-diagnosis, World Health Organization, 2016). A total percentage of 22.1% (19 out of 86 individuals) had a clinical diagnosis of an anxiety disorder co-occurring to autism (see Supplementary Table S1 for further details).

Participants in the non-autistic group were recruited by contacting participants of former studies, sending out letters to people in Dresden (information from the residents’ registration office), advertising the study privately with flyers and on *Facebook* and *Ebay Kleinanzeigen*, by contacting staff of the University Hospital and listing the study on the experimental server of the Technische Universität Dresden (TUD) and the TUD medical school. In addition, some of the non-autistic participants with ANX were recruited with the help of training institutes for psychotherapy. Participants without anxiety disorder in this group were screened via a checklist or brief interview and were only included if they reported no neurological and/or psychological disorder, no autistic first-degree relatives and no recurring use of illegal substances. Participants were, however, included in the non-autistic group if they reported a former or current anxiety disorder (ANX). Of the 13 participants reporting such diagnoses, no further co-occurring psychiatric diagnoses were reported by 7, while 4 reported one co-occurring diagnosis and 2 reported two or more (see Supplementary Table S1 for further information on co-occurring diagnoses of ANX participants). Full-Scale Intelligence Quotient (FIQ) was not available for individuals in the non-autistic group.

### Measures

Participants completed the German versions of the following questionnaires, with higher scores indicating the greater expression of relevant traits:

The *Autism Spectrum Quotient* (AQ; Freitag et al., 2007) is widely used to quantify autistic-like traits with 50 items. It is a dimensional measure sensitive to subclinical autistic traits (Bishop et al., 2004) and has been shown to differentiate between autistic and non-autistic individuals in a German sample (Lehnhardt et al., 2011). Following recommendations based on heterogeneity of factor solutions and inferior psychometric properties of the subscale scores (Hoekstra et al., 2008; Stevenson & Hart, 2017), only the total scale is used as a continuous measure of autistic-like traits in this study. In the current sample, the AQ demonstrated excellent internal consistency in both groups (Cronbach’s alphas: autistic = 0.91; non-autistic = 0.81).

ER was measured using the *Emotion Regulation Questionnaire* (ERQ; Abler & Kessler, 2009), which has been used previously in autistic samples (Cai et al., 2018; Samson et al., 2012). The ERQ captures the use of maladaptive suppression (ERQs) and adaptive reappraisal (ERQr) strategies with a total of 10 items on each of 2 relevant subscales. Following Cai et al., a ratio of ER strategy use was utilised for further analyses, calculated by dividing suppression scores by reappraisal scores. In the current sample, both the reappraisal and suppression subscales demonstrated good internal consistency in both groups (Cronbach’s alphas: autistic reappraisal = 0.85; non-autistic reappraisal = 0.79; autistic suppression = 0.74; non-autistic suppression = 0.81).

IU was measured by the 18-item *Intolerance of Uncertainty Scale* (IU-18; Gerlach et al., 2008), which has been shown to have good psychometric properties in German samples. Because of varying factor solutions that were found in versions in different languages (Buhr & Dugas, 2002; Carleton et al., 2007; Freeston et al., 1994), only the total score was used. In the current sample, the scale demonstrated excellent internal consistency in both groups (Cronbach’s alpha: autistic = 0.93; non-autistic = 0.93).

The *Toronto Alexithymia Scale* (TAS-20; Bach et al., 1996) was utilised to examine ALX with 20 items. It has been validated in a sample of autistic adults (Berthoz & Hill, 2005). Because of the differential factor structure found in a German general population sample (Franz et al., 2008), only the total score was used. In the current sample, the scale demonstrated excellent internal consistency in both groups (Cronbach’s alpha: autistic = 0.88; non-autistic = 0.87).

To measure SPD, the *Glasgow Sensory Questionnaire* (GSQ; Robertson & Simmons, 2013) was used. The GSQ was originally developed to investigate the relationship between autistic-like traits and sensory functioning in the general population. It has been used previously in samples of autistic adults (e.g. Hwang et al., 2019) and contains 42 items. Scores can be calculated for the subscales of hypo- and hyper-sensitivity in seven modalities. We only utilised the total score. Because a German version was not available, a translation was developed for this study with permission of the authors. The validation of this translation with a student sample is currently in revision (Zeisel et al., in press). In the current sample, the scale demonstrated excellent internal consistency (Cronbach’s alphas: autistic = 0.93; non-autistic = 0.86).

Anxiety was measured using the *Liebowitz Social Anxiety Scale* (LSAS; Stangier & Heidenreich, 2003) and the *Hospital Anxiety and Depression Scale* (HADS, Herrmann-Lingen et al., 2011). The LSAS asks about fear and avoidance in 24 interactional and performance situations during the last 7 days. The scale has good psychometric properties and is an adequate tool for the screening for social anxiety disorder (Rytwinski et al., 2009). Moreover, it has been used repeatedly in autistic samples (Spain et al., 2018). Here, only the total score was used. In the current sample, the internal consistency of the scale was excellent (Cronbach’s alphas: autistic = 0.96; non-autistic = 0.96). The anxiety scale of the Hospital Anxiety and Depression Scale (HADSa), which contains seven items, was used to measure generalised anxiety. The HADS was created to measure depression and anxiety in clinical settings. It has been validated for use in autistic samples (Uljarević et al., 2018), and in the current sample, the anxiety subscale demonstrated excellent internal consistency in both groups (Cronbach’s alphas: autistic = 0.80; non-autistic = 0.81).

### Procedure

Data on the seven questionnaires was collected as part of a wider project concerning the improvement of anxiety diagnostics for autistic individuals. Participants, therefore, answered a total of 12 questionnaires either online, via LimeSurvey (Schmitz, 2012), or at home via pen and paper. Completion of the questionnaires required approximately 60 min, and participants were compensated for their time with €10. Informed consent was acquired from each participant, and all study procedures adhered to the declaration of Helsinki and were approved by the ethics committee of TUD (ethical approval code: EK 356092018).

### Data analysis

Statistical analyses were performed using IBM SPSS Statistics 28 and SPSS AMOS 27 Graphics. For all analyses, raw scores of the measures were used. Less than 1% of data were missing, and missing item values were imputed according to the corresponding questionnaire manuals, replaced with the mean item value of the available responses or by generating a corrected total score. Examination of descriptive statistics demonstrated a normal distribution of scores for both groups on the AQ, GSQ, IU and TAS measures. The ERQ ratio score and HADS anxiety score were positively skewed in both groups, as was the LSAS score for the non-autistic group. Differences between groups on all measures were, therefore, analysed with chi-square tests, *t*-tests and Mann–Whitney *U*-tests as appropriate. Pearson’s correlations were used to examine the relationships among observed variables.

**Figure 2.**
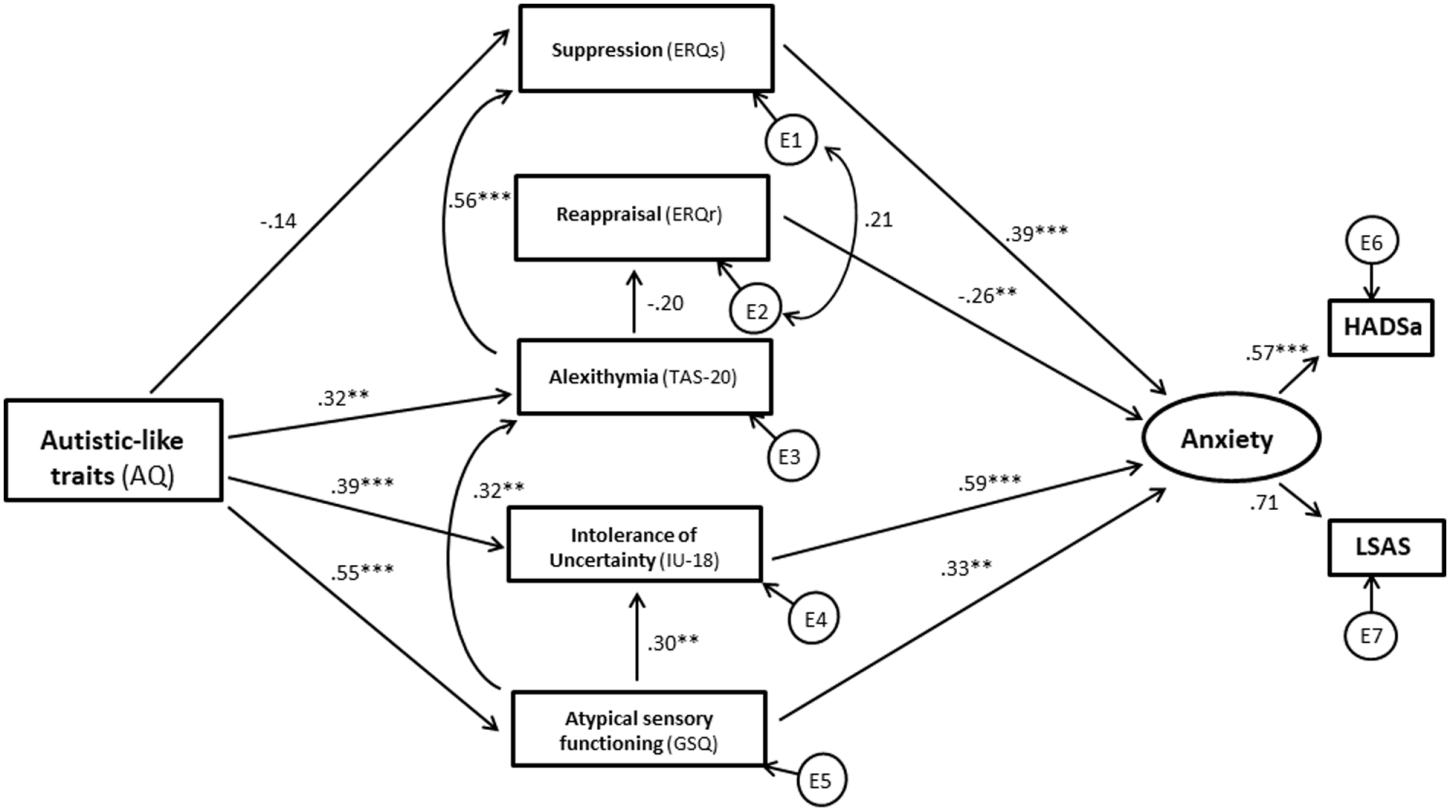
Results of structural equation modelling (autistic group). *Note.* Standardised regression coefficients for the modified model (autistic group). Significance level was determined by bootstrapping procedures and is indicated by asterisks. Rectangles show observed variables and ovals latent variables. Error terms are omitted to aid clarity. *p < .05; **p < .01; ***p < .001.

**Figure 3.**
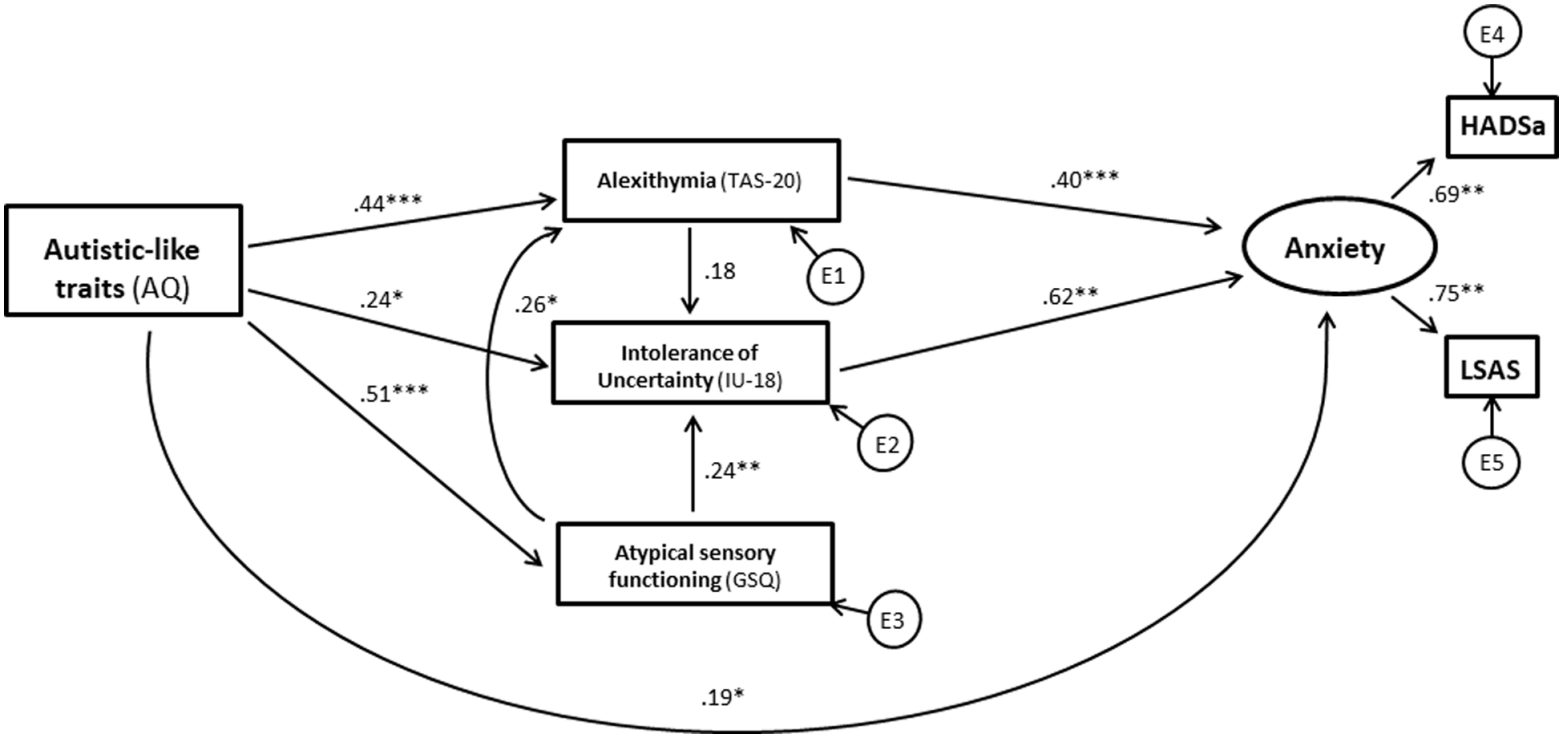
Results of structural equation modelling (non-autistic group). *Note.* Standardised regression coefficients for the modified model (non-autistic group). Significance level was determined by bootstrapping procedures and is indicated by asterisks. Rectangles show observed variables and ovals latent variables. Error terms are omitted in terms of clarity. *p < .05; **p < .01; ***p < .001.

For the standard error of the mean (SEM) analysis, the predicted interrelations set out in Figure 1 above were first examined across both groups combined, using the maximum-likelihood approach and additional bootstrapping procedures.. Autism was treated as a continuous variable in this analysis through AQ scores and a latent variable for *anxiety* was created using the HADSa and LSAS total scores. Similarly to Maisel et al. (2016), we chose anxiety questionnaires measuring different facets of anxiety. Both measures were moderately to highly correlated in both groups (see Table 3). Regarding model fit in SEM, a non-significant chi-square (χ^2^) *p*-value would indicate a good fit between the predicted model and observed data. Because of the non-normal distribution of the ERQ ratio scores, a corrected *p*-value for χ^2^ was computed via Bollen–Stine bootstrap (Bühner, 2006) indicating bad fit, *p* = 0.000 (5000 samples). For the additional parameters, model fit is regarded acceptable when χ^2^/*df* ⩽ 2.5, comparative fit index (CFI) ⩾ 0.90 (Homburg & Baumgartner, 1995), root mean square error of approximation (RMSEA) ⩽ 0.08 (Brown & Cudeck, 1993), PCLOSE > 0.05 and standardised root mean square residual (SRMR) ⩽ 0.08 (Hu & Bentler, 1999). As set out in detail in section ‘Structural equation modelling’ in section ‘Results’, the predicted model did not fit the data across the entire sample and further analyses were, therefore, carried out using a data-driven approach to adjust the predicted model to achieve adequate fit to the data. Modification of the model for the entire sample did not result in a model with adequate fit. Therefore, the model was examined for each group separately. When model fit was not adequate, SPSS AMOS suggested modifications to the model. We performed these modifications stepwise evaluating model fit after every step considering chi-square exact-fit test, CFI, RMSEA and the SRMR. Specifically, suggested pathways were added and some non-significant and low-loading pathways or variables were deleted until adequate model fit was achieved (Rudolf & Müller, 2020). For visualisation of the performed changes, compare Figures 1 and 2 for the autistic group and Figures 1 and 3 for the non-autistic group and refer to section ‘Results’ for further details. Multivariate normality, assessed with the critical ratio to Mardia’s coefficient (Mardia, 1970), was violated, c.r. > 2.57. Therefore, the bias-corrected percentile method using bootstrap (5000 samples) as well as the corrected *p*-value for *χ*^2^ via the Bollen–Stine bootstrapping was utilised.

### Community involvement statement

Not applicable.

## Results

### Between-group comparisons

Group comparisons for all dependent variables of interest are set out in Table 2 and confirmed significantly higher levels of anxiety, ER difficulties, ALX, SPD and IU for the autistic compared to the non-autistic group. ER difficulties were related primarily to the reduced use of adaptive reappraisal strategies while no differences between groups were reported in the use of maladaptive suppression (Table 2).

**Table 2.** Descriptive statistics and group comparisons for all key variables of interest.

	Autistic (*n* = 86)		Non-autistic (*n* = 100)						95% CI	
Measure	M	SD	M	SD	Range	*t*(*df*) or *U*(*z*) *	*p*	*d*	Lower	Upper
AQ	32.8	8.9	17.9	7.4	0–50	12.49 (184)	<.001*	1.84	1.49	2.18
ERQ ratio	0.76	0.47	0.60	0.31	0.1–4.7	3397 (2.47)*	.014*	0.42	0.13	0.71
ERQr	23.1	8.3	26.4	6.7	6–42	5372 (2.93)*	.003*	0.44	0.15	0.74
ERQs	15.1	5.9	14.6	5.1	4–28	4055 (0.67)*	.503	0.08	−0.21	0.37
IU-18	56.4	14.9	46.9	15.7	18–90	4.18 (184)	<.001*	0.62	0.32	0.91
GSQ	55.1	24.3	39.2	16.0	0–168	5.19 (143.15)	<.001*	0.79	0.49	1.09
TAS-20	54.7	13.7	45.8	11.4	20–100	4.81 (184)	<.001*	0.71	0.41	1.00
HADSa	7.8	3.9	6.5	4.3	0–21	3344 (2.62)*	.009*	0.33	0.04	0.62
LSAS	60.5	27.3	38.9	26.4	0–144	2404 (5.18)*	<.001*	0.80	0.50	1.12

CI: confidence interval; AQ: Autism Spectrum Quotient; ERQ: Emotion Regulation Questionnaire; ERQr: Emotion Regulation Questionnaire reappraisal; ERQs: Emotion Regulation Questionnaire suppression; IU-18: 18-item Intolerance of Uncertainty Scale; GSQ: Glasgow Sensory Questionnaire; TAS-20: Toronto Alexithymia Scale; HADSa: Hospital Anxiety and Depression Scale; LSAS: Liebowitz Social Anxiety Scale.

*Note*. The range column indicates the possible range of scores for each variable. Cohen’s *d* is included as a measure of effect size including 95% confidence intervals.

* *p* < .05.

### Correlations

Table 3 provides an overview of the Pearson correlation coefficients of associations among measures across both groups combined. With the exception of the association between ERQ and SPD, which was not significant, the data confirm moderate to strong interrelations among all variables of interest. Table 3 also sets out the relevant correlations for each group separately and the pattern of associations was broadly similar within both groups. Different was that associations between HADS anxiety and AQ, ERQ and TAS were only significant in the non-autistic group, as were associations between ERQ and IU-18 and AQ.

**Table 3.** Pearson’s correlation coefficients of study variables.

Combined (*n* = 186)						
Measure	1	2	3	4	5	6
1. AQ	–					
2. ERQ	0.29*	–				
3. IU-18	0.56*	0.20*	–			
4. GSQ	0.61*	0.12	0.53*	–		
5. TAS-20	0.59*	0.44*	0.46*	0.55*	–	
6. HADSa	0.35*	0.25*	0.61*	0.49*	0.41*	–
7.LSAS	0.61*	0.33*	0.65*	0.54*	0.61*	0.52*
Autistic (*n* = 86)						
Measure	1	2	3	4	5	6
1. AQ	–					
2. ERQ	0.12	–				
3. IU-18	0.56*	0.04	–			
4. GSQ	0.55*	0.01	0.52*	–		
5. TAS-20	0.49*	0.39*	0.37*	0.49*	–	
6. HADSa	0.21	0.16	0.51*	0.48*	0.28	–
7. LSAS	0.47*	0.30*	0.58*	0.47*	0.49*	0.43*
Non-autistic (*n* = 100)						
Measure	1	2	3	4	5	6
1. AQ	–					
2. ERQ	0.37*	–				
3. IU-18	0.47*	0.29*	–			
4. GSQ	0.51*	0.12	0.46*	–		
5. TAS-20	0.57*	0.44*	0.44*	0.48*	–	
6. HADSa	0.44*	0.31*	0.66*	0.49*	0.50*	–
7. LSAS	0.57*	0.26	0.46*	0.47*	0.62*	0.56*

AQ: Autism Spectrum Quotient; ERQ: Emotion Regulation QuestionnaireIU-18: 18-item Intolerance of Uncertainty Scale; GSQ: Glasgow Sensory Questionnaire; TAS-20: Toronto Alexithymia Scale; HADSa: Hospital Anxiety and Depression Scale; LSAS: Liebowitz Social Anxiety Scale.

*Note.* Total scores were utilised for all measures.

* *p* < 0.007 (Bonferroni-adjusted).

### Structural equation modelling

As noted above, an initial SEM analysis across both groups combined to test the predicted model set out in Figure 1 did not achieve adequate model fit (χ^2^(12) = 103.376, *p* = .000, χ^2^/*df* = 8.615; RMSEA = 0.203, PCLOSE = 0.000; CFI = 0.839; SRMR = 0.113), even following data-driven modifications to the predicted model. Further analyses, therefore, tested the predicted model against the data in each group separately.

#### Autistic group SEM analysis

Overall model fit for the autistic sample was not adequate (χ^2^(12) = 49.031, *p* = .000 (Bollen–Stine *p* = .000), χ^2^/*df* = 4.086; RMSEA = 0.191, PCLOSE = 0.000; CFI = 0.812; SRMR = 0.111). However, following stepwise data-driven additions of pathways from SPD directly to anxiety and ALX, and directly from AQ to IU, and the removal of our predicted pathway from ALX to IU, adequate model fit was achieved (compare Figures 1 and 2 for changes to the model) (χ^2^(11) = 16.196, *p* = .134 (Bollen–Stine *p* = .203), χ^2^/*df* = 1.472; RMSEA = 0.075, PCLOSE = 0.270; CFI = 0.974; SRMR = 0.065). All pathways in this modified model were in the expected directions. Autistic-like traits significantly predicted more ALX (β = 0.32, *p* = .005), IU (β = 0.39, *p* < .001) and SPD (β = 0.55, *p* < .001). ALX significantly predicted a higher ER ratio (β = 0.39, *p* = .001). ER ratio significantly predicted more anxiety (β = 0.34, *p* < .001). IU significantly predicted more anxiety (β = 0.61, *p* < .001), so did SPD (β = 0.40, *p* = .004). Also, SPD significantly predicted more ALX (β = 0.32, *p* = .007) and more IU (β = 0.30, *p* = .006). Multiple squared correlations of endogenous variables are provided in Supplementary Table S2, and standardised estimates of direct, indirect and total effects with corresponding 95% bootstrap confidence intervals are provided in Supplementary Table S3.

Standardised regression weights for specific indirect effects are reported in Supplementary Table S4. Briefly, the indirect effect of autistic-like traits on anxiety via IU was significant (β = 0.24, *p* < .001) as well as the effect of SPD on anxiety via IU (β = 0.19, *p* = .003). The effect of autistic-like traits on anxiety via SPD was also significant (β = 0.22, *p* = .002) as was the indirect effect of ALX on anxiety via ER ratio (β = 0.13, *p* < .001).

#### Non-autistic group SEM analysis

Overall model fit for the non-autistic sample was not adequate (χ^2^(12) = 63.024, *p* = 0.000 (Bollen–Stine *p* = .001), χ^2^/*df* = 5.252; RMSEA = 0.207, PCLOSE = 0.000; CFI = 0.816; SRMR = 0.134) nor could adequate fit be achieved through stepwise data-driven additions or deletions of pathways. However, when the factors autistic-like traits and SPD were removed from the model, and a model was tested that only included well-established risk factors for anxiety in the general population (IU, ALX and ER), the model set out in Figure 3 could be derived by adding a direct pathway from ALX to anxiety (compare Figures 1 and 3 to inspect the changes to the model) (χ^2^(3) = 4.520, *p* = .210 (Bollen–Stine *p* = .595), χ^2^/*df* = 1.507; RMSEA = 0.072, PCLOSE = 0.307; CFI = 0.992; SRMR = 0.026).

Multiple squared correlations of endogenous variables are provided in Supplementary Table S5. Supplementary Table S6 sets out the standardised estimates of direct, indirect and total effects with corresponding 95% bootstrap confidence intervals for the above model. In this modified model for the non-autistic group, ALX significantly predicted more anxiety (β = 0.49, *p* < .001) as well as greater levels of IU (β = 0.39, *p* = .002). IU significantly predicted more anxiety (β = 0.69, *p* < .001). The pathways from ER ratio to IU (β = 0.12, *p* = .204) and to anxiety (β = −0.02, *p* = .855) were non-significant. The indirect effect of ALX on anxiety via IU was significant (β = 0.27, *p* = .001), while all other specific indirect effects were non-significant (for all specific indirect effects see Supplementary Table S7).

## Discussion

This study investigated the role of SPD, ALX, IU and ER in the relationship between autistic-like traits and anxiety in autistic and non-autistic adults with average intelligence. In line with existing evidence (Hwang et al., 2019; Kinnaird et al., 2019; Lehnhardt et al., 2011; Samson et al., 2012; Takayama et al., 2014), participants in the autistic group scored significantly higher on measures for autistic-like traits, ALX, IU, SPD and anxiety. The autistic group also demonstrated a more maladaptive pattern of ER skills that was characterised primarily by significantly lower reported use of reappraisal relative to suppression strategies compared to the non-autistic group. No significant difference was observed between groups in the use of suppression strategies per se, which some previous studies suggest are sometimes used more frequently in autism (Samson et al., 2012). As Cai et al. (2018) suggest, however, the relative use of adaptive versus maladaptive strategies often has greater implications for mental health than the absolute degree to which either strategy might be used, which was supported by the current findings in terms of the correlation between an ER ratio score and measures of anxiety. More broadly, the pattern of correlations among measures also confirmed previous observations of significant interrelations among measures of autistic-like traits, SPD, ALX, IU and ER difficulties that this study sought to shed further light on. Specifically, our primary aim was to test a specific model of these interrelations, which predicts that IU and ER difficulties constitute relatively proximal causes of anxiety in autism that mediate the impact of ALX and SPD.

When testing the predicted model against the combined data across both groups, no adequate fit could be achieved, even when adjusting model paths based on data-driven principles. When the model was tested against the autistic group data alone, however, adequate fit was achieved after some data-driven adjustments. The resulting model confirmed that IU, ER and SPD play a relatively central role in anxiety in autism. In addition, SPD and ALX contribute to anxiety through respective associations with IU and ER difficulties. This pattern fits well with an emerging consensus view about the role of IU and ER in anxiety in autism (Boulter et al., 2014; South & Rodgers, 2017) and further suggests that each of these factors can contribute independently to experiences of anxiety. The findings also support ongoing developments in clinical practice, where interventions focussed on building tolerance of uncertainties (Rodgers et al., 2017, 2018) and ER skills (e.g. Conner et al., 2019; Weiss et al., 2018) are often effective in the treatment of anxiety in autism.

The required adjustments to the model to achieve adequate fit have important implications for understanding the role of SPD and ALX. Based on a model by South and Rodgers (2017), and subsequent findings (Moore et al., 2021; Ozsivadjian et al., 2021), we predicted that ALX would contribute to anxiety not only by increasing ER difficulties but also by rendering internal emotional states confusing and, thereby, contributing to IU. Our model did not support this prediction, suggesting instead that ALX contributes relatively independently to anxiety only via associations with ER difficulties. By contrast, SPD did not only impact upon anxiety through indirect influences on IU (Hwang et al., 2019; Wigham et al., 2015) but also through an independent direct pathway, which supports findings by Pickard et al. (2020). Interestingly, the study by Pickard et al. (2020) was concerned with predictors of social anxiety, whereas the studies by Hwang et al. (2019) and Wigham et al. (2015) examined predictors of more generalised anxiety. In this study, we derived a latent anxiety factor from measures of both generalised and social anxiety, and it is, therefore, possible that SPD plays a relatively greater role in social rather than more generalised forms of anxiety in autism. This suggestion aligns with evidence, which suggests that autistic individuals often experience social interactions as difficult because of associated sensory processing demands (Hilton et al., 2010). To examine this further is a task for future research.

A further model adjustment that was required to achieve adequate fit and that has interesting implications for the literature is the addition of a pathway from SPD to ALX. Specifically, growing literature suggests that ALX is closely associated with aspects of interoception, which describes the processes involved in sensing internal physiological states such as changes in heart rate or arousal (Brewer et al., 2016). In addition, evidence suggests that autism may be associated with certain interoceptive difficulties (DuBois et al., 2016) that are closely linked to ALX (Shah et al., 2016), anxiety (Garfinkel et al., 2016; Palser et al., 2018) and SPD (Proff et al., 2021). Within the context of this wider literature, the findings of this study may, therefore, suggest that SPD contribute to ALX in autism by altering relevant underlying interoceptive processes and that ALX in turn contributes to anxiety by compromising adaptive patterns of ER. Clinically, the findings suggest that interventions targeting SPD could potentially reap benefits for anxiety in autism. Sensory-based interventions are already widely used, but the evidence base for their effectiveness is currently relatively weak, owing partly to methodological weaknesses in relevant trials and a lack of consensus regarding conceptualisations of SPD in autism as well as the desired outcomes of interventions (see, for example, Case-Smith et al., 2015).

The final model adjustment that was necessary for the data of the autistic group was the introduction of a direct path from autism-related traits to IU, which suggests that IU is not exclusively linked to SPD in autism. Conceptually, it is linked to insistence on sameness and a recent study reported increased anxiety rates in autistic children that were preceded by elevated levels of insistence on sameness behaviour 1–2 years earlier (Baribeau et al., 2022). In addition, previous studies have shown that factors such as cognitive flexibility may also play an important role in the relationship between the core clinical characteristics of autism and IU (Ozsivadjian et al., 2021), and other studies point to a role of Theory of Mind (ToM) difficulties in the expression of anxiety in autism (Lei & Ventola, 2018). Further cognitive differences that have recently been suggested to play a role in the development of anxiety in autism are reduced predictive processing and ‘black and white thinking’ (Stark et al., 2021). Thus, the model tested in this study is unlikely to represent all of the factors that contribute to increased levels of anxiety in autism.

Turning now to the finding that the predicted model could not be fit to the data across both groups combined, it is interesting that no adequate model fit could be achieved when the data were considered from the non-autistic group alone. In the context of the model that was ultimately derived for the autistic group, this may suggest that the mechanisms involved in anxiety in autism are, at least to some extent, distinct from those involved in anxiety in the general population. The finding that anxiety can often manifest differently in autism than in the general population supports this suggestion. For example, following detailed clinical interviews with parents of autistic children, Kerns et al. (2014) found that 15% of parents described patterns of anxiety in their children that would not fall under traditional definitions of anxiety, and a further 30% described combinations of such ‘atypical’ presentations of anxiety together with more typical presentations. Often, the autism-specific expression of anxiety reported in this study was related to worries about changes in routines (i.e. IU) or fears of particular sensory stimuli (i.e. SPD), which are not typically reported as sources of anxiety in the general population. In addition, factors such as cognitive inflexibility and ToM difficulties may also play relatively unique roles in the expression of anxiety in autism as compared to the general population (Lei & Ventola, 2018; Ozsivadjian et al., 2021). In the context of this wider literature, it is, therefore, perhaps not surprising to observe distinct patterns of interrelationships among relevant constructs.

Based on the considerations above, we examined the data in the non-autistic group after removing autism-related traits and SPD as factors in the model, focussing instead on only those risk factors (IU, ALX and ER) that are well established to play a role in anxiety in the general population (Aldao et al., 2016; Carleton et al., 2012; Kranzler et al., 2016). With only these factors in the model, adequate fit was achieved, demonstrating that ALX and IU exert independent direct influences on anxiety. Although ALX was also associated with ER in this model, ER did not significantly contribute to anxiety either directly or indirectly. This observation is somewhat unexpected given the consistent evidence that links ER to anxiety and other mental health difficulties (e.g. Aldao et al., 2016). However, to the best of our knowledge, no previous studies have examined the combined contributions of ALX, IU and ER to individual differences in anxiety in the general population. In such general population samples, ALX and IU may play a relatively more important role in anxiety than ER. In this context, it is also relevant to note that the distribution of the ER ratio score, as well as both the HADS and LSAS anxiety scores, was skewed in our non-autistic group. The implications of the observations for models of clinical levels of anxiety are, therefore, somewhat unclear, and future studies should attempt to compare predictors of anxiety in autistic samples to the predictors of similar levels of anxiety in non-autistic anxious individuals.

When considering our results, one should consider the transdiagnostic nature of our sample. Including individuals with co-occurring disorders in the autistic group and anxiety disorders in the non-autistic group will potentially increase the generalisability of our findings. In addition, for the same reason, we included a sample with a large age range. Therefore, age may have impacted on the results. However, co-occurring neurological conditions whose risk increases with age are very unlikely in the current sample since participants were screened for those prior participation and only 13 participants in the total sample were 60 years of age and above.

Before drawing this discussion to a close, it is important to acknowledge a number of potential limitations in this study. First, the study did not include autistic individuals with significant language impairments and/or learning disabilities and, therefore, the findings may not generalise across the autism spectrum, particularly as some evidence suggests that higher intellectual functioning may be associated with greater levels of anxiety in autism (e.g. Nimmo-Smith et al., 2020). Having said that, a recent study of autistic adults with ID found that IU and ER difficulties constituted independent mediators of the relationship between autism-related behaviours and anxiety (Sáez-Suanes et al., 2020), suggesting that the model presented in this article may generalise across the autism spectrum. Further studies involving representative samples are necessary, however, to further examine this issue. A second limitation of the study is that it relied exclusively on self-report measures to capture all constructs of interest, none of which were developed and validated specifically for autistic respondents. Although the internal consistencies of all measures used in this study were very good in both groups within the current sample, and all measures have been used in previous studies of anxiety in autism, there is a potential danger of missing autism-specific characteristics of anxiety (see, for example, Kerns et al., 2014). Recently, the first autism-specific self-report questionnaire for anxiety has been developed and validated for adult participants (Rodgers et al., 2020), which will help to address this concern in future research. In addition, however, it will also be important to develop more behavioural measures of key constructs of interest in order to bridge our understanding of the relationship between internalising and externalising symptoms of anxiety. Such a comprehensive measurement of anxiety will also be important for translating theoretical models such as the one described here into relevant interventions effectively. A third limitation concerns the sample size of this study, which may be considered as relatively small. Our sample size calculations were guided by previous literature. Maisel et al. (2016) included 76 autistic adults and 76 non-autistic individuals. In addition, we followed the general recommendation of 10 participants for every parameter (e.g. Hoe, 2008). Despite our greatest efforts in recruitment, we did not achieve this fully for the autistic group. Therefore, with our data, it was not possible to examine further variables or add additional pathways. The aim of this study was to establish and test a basic model of the development of anxiety in autism. One of the next steps could be to expand the current model to include returning pathways from anxiety to assess the impact of anxiety on autism-related symptoms. As mentioned in the introduction, Neil et al. (2016) have shown that anxiety can also be considered a mediator of the relationship between IU and SPD. To examine this and other additional pathways is a task for future research. Furthermore, the question arises whether different models of anxiety can be derived when considering different types of anxiety separately such as social and general anxiety. We chose a rather broad construct of anxiety including social and general anxiety in our latent variable for our basic model, which was done according to the previous literature such as Maisel et al. (2016). Future research testing larger samples could analyse and compare separate models for different anxiety types.

A final limitation is that we implemented a variable-centred analysis in this study. The above reported associations may not apply to subgroups or at the individual level, that is, in some individuals IU or SPD may be the most relevant factor in driving their anxiety. However, the investigation of anxiety models in autistic subgroups could be a topic of future studies.

In terms of the possible clinical implications of the current work, the findings are in line with growing evidence which suggests that interventions for anxiety in autism that target ER strategy use, ALX and IU generally proof to be effective. For example, recent reviews of cognitive behavioural therapies (CBTs; Spain et al., 2015) and mindfulness-based therapies (Cachia et al., 2016) suggest that they are effective in alleviating anxiety in autism. In addition, a newly developed intervention that utilises CBT and mindfulness-based practices to target IU specifically is yielding promising results as a means of managing anxiety in autism (Rodgers et al., 2018). Such work demonstrates the importance of tailoring interventions specifically for the autistic community, and the findings of this study may help by highlighting the importance that SPD may also play in this context. SPDs have for long been a target of interventions but rarely with a specific focus on anxiety as outcomes. For example, sensory integration therapy is often used to target broad functional skills and adaptive behaviours rather than anxiety and the evidence for its effectiveness is rather mixed (see Case-Smith et al., 2015; Lang et al., 2012). However, there are other types of interventions that may be fruitfully adapted and/or integrated with approaches such as CBT or mindfulness-based interventions to target anxiety in autism, such as systematic desensitisation (Koegel et al., 2004). In general, interventions targeting several factors influencing anxiety in combination will potentially be more beneficial rather than targeting each factor in isolation.

## Supplemental Material

sj-docx-1-aut-10.1177_13623613231177777 – Supplemental material for Examining a model of anxiety in autistic adults Supplemental material, sj-docx-1-aut-10.1177_13623613231177777 for Examining a model of anxiety in autistic adults by Saskia Riedelbauch, Sebastian B Gaigg, Tobias Thiel, Veit Roessner and Melanie Ring in Autism
