# Supplementary material for: Examining a model of anxiety in autistic adults
Source: Autism. 2023 Jun 16;28(3):565–79. doi: 10.1177/13623613231177777 (PMC10913331; doi:10.1177/13623613231177777)
Supplement: sj-docx-1-aut-10.1177_13623613231177777 – Supplemental material for Examining a model of anxiety in autistic adults [file sj-docx-1-aut-10.1177_13623613231177777.docx]

Supplementary Material

Table S1

*Quantity of Additional Mental, Behavioral and Neurodevelopmental Disorders (Lifetime F-diagnoses) of Autistic and ANX Subjects*

|  | F1 | F2 | F3 | F4 | F5 | F6 | F7 | F8 | F9 |
| --- | --- | --- | --- | --- | --- | --- | --- | --- | --- |
| Autistic (n=86) |  |  |  |  |  |  |  |  |  |
| No diagnosis | 83 | 84 | 55 | 67 | 84 | 74 | 85 | 68 | 73 |
| Diagnosis | 3 | 2 | 31 | 19 | 2 | 12 | 1 | 18 | 13 |
| ANX (n=13) |  |  |  |  |  |  |  |  |  |
| No diagnosis | 13 | 13 | 7 | 9 | 13 | 13 | 13 | 13 | 13 |
| Diagnosis | 0 | 0 | 6 | 4 | 0 | 0 | 0 | 0 | 0 |

*Note*. Diagnoses according to ICD-10 (World Health Organization, 2016). Autism diagnosis of autistic subjects and primary anxiety diagnosis of ANX subjects are not included in the count. F1: Mental and behavioral disorders due to psychoactive substance use. F2: Schizophrenia, schizotypal, delusional, and other non-mood psychotic disorders. F3: Affective disorders. F4: Anxiety, dissociative, stress-related, somatoform and other nonpsychotic mental disorders. F5: Behavioral syndromes associated with physiological disturbances and physical factors. F6: Disorders of adult personality and behavior. F7: Intellectual disabilities. F8: Pervasive and specific developmental disorders. F9: Behavioral and emotional disorders with onset usually occurring in childhood or adolescence.

Table S2

*Squared Multiple Correlations for Endogenous Variables in Modified Model for the Autistic Group*

|  |  |  |  | 95% Confidence Interval | |
| --- | --- | --- | --- | --- | --- |
| Parameter | *R^2^* | *SE* | *p* | Lower | Upper |
| SPD | .30 | .09 | <.001 | .14 | .48 |
| ALX | .31 | .09 | .001 | .14 | .48 |
| ER | .15 | .05 | .001 | .06 | .27 |
| IU | .38 | .09 | .001 | .20 | .54 |
| Anxiety (LSAS) | .48 | .08 | .002 | .27 | .62 |
| Anxiety  (HADSa) | .36 | .11 | .001 | .14 | .55 |

*Note*. Standard-errors, *p*-values and confidence intervals were computed using bootstrap (5000 samples). *R^2^* displays the proportion of variance of a parameter that is explained by its predictors. *R^2^* of LSAS and HADSa imply that there are additional variables influencing anxiety.

Table S3

*Standardized Estimates of SEM-Based Mediation Analyses for the Final Model of the Autistic Group*

| Direct effects |  |  |  | 95% Confidence Interval | |
| --- | --- | --- | --- | --- | --- |
|  | Estimate | *SE* | *p* | Lower | Upper |
| ASD 🡪 SPD | .55 | .08 | <.001 | .38 | .69 |
| ASD 🡪 ALX | .32 | .10 | .005 | .10 | .50 |
| ASD 🡪 IU | .39 | .09 | <.001 | .21 | .57 |
| SPD 🡪 ALX | .32 | .11 | .007 | .09 | .52 |
| SPD 🡪 IU | .30 | .10 | .006 | .10 | .48 |
| SPD 🡪 Anxiety | .40 | .13 | .004 | .14 | .65 |
| ALX 🡪 ER | .39 | .07 | .001 | .25 | .52 |
| ER 🡪 Anxiety | .34 | .10 | <.001 | .15 | .54 |
| IU 🡪 Anxiety | .61 | .12 | <.001 | .38 | .84 |
| Anxiety 🡪 LSAS | .69 | .06 | .002 | .52 | .78 |
| Anxiety 🡪 HADSa | .60 | .09 | .001 | .38 | .74 |
| Indirect effects |  |  |  | 95% Confidence Interval | |
|  | Estimate | *SE* | *p* | Lower | Upper |
| ASD 🡪 ALX | .17 | .07 | .004 | .06 | .33 |
| ASD 🡪 ER | .19 | .05 | <.001 | .10 | .29 |
| ASD 🡪 IU | .17 | .06 | .003 | .07 | .29 |
| ASD 🡪 Anxiety | .62 | .07 | <.001 | .48 | .74 |
| ASD 🡪 LSAS | .43 | .07 | <.001 | .29 | .57 |
| ASD 🡪 HADSa | .37 | .07 | <.001 | .24 | .52 |
| SPD 🡪 ER | .12 | .05 | .005 | .04 | .23 |
| SPD 🡪 Anxiety | .23 | .07 | <.001 | .11 | .40 |
| SPD 🡪 LSAS | .43 | .07 | <.001 | .28 | .57 |
| SPD 🡪 HADSa | .37 | .09 | <.001 | .20 | .56 |
| ALX 🡪 Anxiety | .13 | .05 | <.001 | .06 | .25 |
| ALX 🡪 LSAS | .09 | .04 | <.001 | .04 | .18 |
| ALX 🡪 HADSa | .08 | .03 | <.001 | .04 | .15 |
| ER 🡪 LSAS | .23 | .07 | .001 | .10 | .38 |
| ER 🡪 HADSa | .20 | .06 | <.001 | .09 | .33 |
| IU 🡪 LSAS | .42 | .10 | <.001 | .24 | .62 |
| IU 🡪 HADSa | .36 | .09 | <.001 | .21 | .55 |

| Total effects |  |  |  | 95% Confidence Interval | |
| --- | --- | --- | --- | --- | --- |
|  | Estimate | *SE* | *p* | Lower | Upper |
| ASD 🡪 SPD | .55 | .08 | <.001 | .38 | .69 |
| ASD 🡪 ALX | .49 | .08 | .001 | .31 | .64 |
| ASD 🡪 ER | .19 | .05 | <.001 | .10 | .29 |
| ASD 🡪 IU | .56 | .08 | <.001 | .38 | .69 |
| ASD 🡪 Anxiety | .62 | .07 | <.001 | .48 | .74 |
| ASD 🡪 LSAS | .43 | .07 | <.001 | .29 | .57 |
| ASD 🡪 HADSa | .37 | .07 | <.001 | .24 | .52 |
| SPD 🡪 ALX | .32 | .11 | .007 | .09 | .52 |
| SPD 🡪 ER | .12 | .05 | .005 | .04 | .23 |
| SPD 🡪 IU | .30 | .10 | .006 | .10 | .48 |
| SPD 🡪 Anxiety | .63 | .11 | <.001 | .41 | .81 |
| SPD 🡪 LSAS | .43 | .07 | <.001 | .28 | .57 |
| SPD 🡪 HADSa | .37 | .09 | <.001 | .20 | .56 |
| ALX 🡪 ER | .39 | .07 | .001 | .25 | .52 |
| ALX 🡪 Anxiety | .13 | .05 | <.001 | .06 | .25 |
| ALX 🡪 LSAS | .09 | .04 | <.001 | .04 | .18 |
| ALX 🡪 HADSa | .08 | .03 | <.001 | .04 | .15 |
| ER 🡪 Anxiety | .34 | .10 | <.001 | .15 | .54 |
| ER 🡪 LSAS | .23 | .07 | .001 | .10 | .38 |
| ER 🡪 HADSa | .20 | .06 | <.001 | .09 | .33 |
| IU 🡪 Anxiety | .61 | .12 | <.001 | .38 | .84 |
| IU 🡪 LSAS | .42 | .10 | <.001 | .24 | .62 |
| IU 🡪 HADSa | .36 | .09 | <.001 | .21 | .55 |
| Anxiety 🡪 LSAS | .69 | .06 | .002 | .52 | .78 |
| Anxiety 🡪 HADSa | .60 | .09 | .001 | .38 | .74 |

*Note*. Only estimates > 0 are listed in this table. Standard-errors, *p*-values and confidence intervals were computed using bootstrap with the bias-corrected percentile method (5000 samples).

Table S4

*Standardized Specific Indirect Effects Observed in the Modified Model for the Autistic Group*

| Pathway | Indirect Effect |
| --- | --- |
| ASD 🡪 IU 🡪 Anxiety | .24*** |
| ASD 🡪 SPD 🡪 Anxiety | .22** |
| ASD 🡪 SPD 🡪 IU 🡪 anxiety | .17** |
| ASD 🡪 ALX 🡪 ER 🡪 anxiety | .12** |
| ASD 🡪 SPD 🡪 ALX 🡪 ER 🡪 anxiety | .17** |
| SPD 🡪 IU 🡪 anxiety | .19** |
| SPD 🡪 ALX 🡪 ER 🡪 anxiety | .12** |
| ALX 🡪 ER 🡪 anxiety | .13*** |

*Note.* Indirect effects were calculated by multiplying the standardized regression weights of corresponding pathways. **p* <.05, ***p* <.01, ****p* <.001, *p*-values were estimated using the bias-corrected percentile method via bootstrapping (5000 samples) with user defined estimands depicting the shown pathway combinations.

Table S5

*Squared Multiple Correlations for Endogenous Variables in the Modified Model for the Non-autistic Group*

|  |  |  |  | 95% Confidence Interval | |
| --- | --- | --- | --- | --- | --- |
| Parameter | *R^2^* | *SE* | *p* | Lower | Upper |
| ER | .19 | .07 | .001 | .08 | .33 |
| IU | .21 | .08 | .001 | .07 | .37 |
| LSAS | .54 | .08 | .001 | .37 | .69 |
| HADSa | .48 | .08 | .002 | .30 | .62 |

*Note*. Standard-errors, *p*-values and confidence intervals were computed using bootstrap (5000 samples). *R^2^* displays the proportion of variance of a parameter that is explained by its predictors. *R^2^* of LSAS and HADS imply that there are additional variables influencing anxiety.

Table S6

*Standardized Estimates of SEM-Based Mediation Analyses for the Final Model of the Non-autistic Group*

| Direct effects |  |  |  | 95% Confidence Interval | |
| --- | --- | --- | --- | --- | --- |
|  | Estimate | *SE* | *p* | Lower | Upper |
| ALX 🡪 ER | .44 | .08 | .001 | .27 | .58 |
| ALX 🡪 IU | .39 | .11 | .002 | .14 | .59 |
| ALX 🡪 Anxiety | .49 | .09 | <.001 | .31 | .67 |
| ER 🡪 IU | .12 | .09 | .204 | -.06 | .30 |
| ER 🡪 Anxiety | -.02 | .11 | .855 | -.22 | .19 |
| IU 🡪 Anxiety | .69 | .07 | <.001 | .55 | .81 |
| Anxiety 🡪 LSAS | .74 | .06 | .001 | .61 | .83 |
| Anxiety 🡪 HADSa | .69 | .06 | .002 | .55 | .79 |
| Indirect effects |  |  |  | 95% Confidence Interval | |
|  | Estimate | *SE* | *p* | Lower | Upper |
| ALX 🡪 IU | .05 | .05 | .18 | -.02 | .16 |
| ALX 🡪 Anxiety | .29 | .08 | .001 | .13 | .45 |
| ALX 🡪 LSAS | .58 | .07 | <.001 | .42 | .71 |
| ALX 🡪 HADSa | .55 | .06 | .001 | .42 | .66 |
| ER 🡪 Anxiety | .09 | .06 | .19 | -.04 | .20 |
| ER 🡪 LSAS | .05 | .10 | .65 | -.16 | .24 |
| ER 🡪 HADSs | .05 | .10 | .65 | -.15 | .25 |
| IU 🡪 LSAS | .51 | .06 | <.001 | .40 | .62 |
| IU 🡪 HADSa | .48 | .07 | .001 | .33 | .60 |
| Total effects |  |  |  | 95% Confidence Interval | |
|  | Estimate | *SE* | *p* | Lower | Upper |
| ALX 🡪 ER | .44 | .08 | .001 | .27 | .58 |
| ALX 🡪 IU | .44 | .10 | <.001 | .23 | .61 |
| ALX 🡪 Anxiety | .79 | .06 | <.001 | .66 | .89 |
| ALX 🡪 LSAS | .58 | .07 | <.001 | .42 | .71 |
| ALX 🡪 HADS | .55 | .06 | .001 | .42 | .66 |
| ER 🡪 IU | .12 | .09 | .20 | -.06 | .30 |
| ER 🡪 Anxiety | .07 | .14 | .65 | -.21 | .35 |
| ER 🡪 LSAS | .05 | .10 | .65 | -.16 | .24 |
| ER 🡪 HADSa | .05 | .10 | .65 | -.15 | .26 |
| IU 🡪 Anxiety | .69 | .07 | <.001 | .55 | .81 |
| IU 🡪 LSAS | .51 | .06 | <.001 | .40 | .62 |
| IU 🡪 HADSa | .48 | .07 | .001 | .33 | .60 |
| Anxiety 🡪 LSAS | .74 | .06 | .001 | .61 | .83 |
| Anxiety 🡪 HADSa | .69 | .06 | .002 | .55 | .79 |

*Note*. Only estimates > 0 are listed in this table. Standard-errors, *p*-values and confidence intervals were computed using bootstrap with the bias-corrected percentile method (5000 samples).

Table S7

*Standardized Specific Indirect Effects Observed in the Modified Model for the Non-autistic Group*

| Pathway | Indirect Effect |
| --- | --- |
| ALX 🡪 IU 🡪 Anxiety | .27** |
| ALX 🡪 ER 🡪 Anxiety | -.01 |
| ALX 🡪 ER 🡪 IU 🡪 Anxiety | .05 |
| ER 🡪 IU 🡪 Anxiety | .09 |

*Note.* Indirect effects calculated by multiplying the standardized regression weights of corresponding pathways. * *p* < .05, ***p* <.01, ****p* <.001, *p*-values were estimated using the bias-corrected percentile method via bootstrapping (5000 samples) with user defined estimands depicting the shown pathway combinations.
